# Supplementary material for: A Personalized Approach to Improve Walking Detection in Real-Life Settings: Application to Children with Cerebral Palsy
Source: Sensors (Basel). 2019 Dec 3;19(23):5316. doi: 10.3390/s19235316 (PMC6928702; doi:10.3390/s19235316)
Supplement: Supplementary file 1 [file sensors-19-05316-s001.pdf]

Figure S1 - Relation between the cadence of the walking bout (detected by FFT) in the laboratory and the 3 temporal thresholds Th2, Th3, Th4:

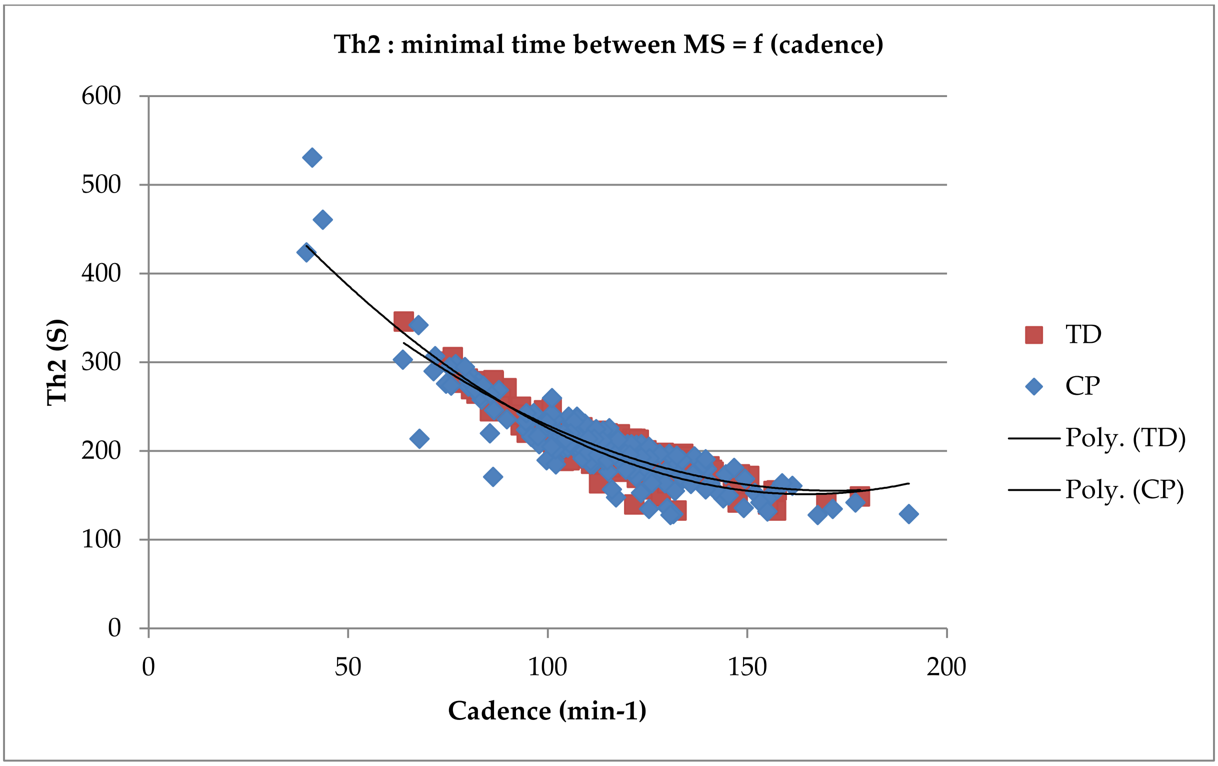

TD : y = $0.0179x^2 - 5.8958x + 636.31$; $R^2 = 0.833$

CP: y = $0.0144x^2 - 4.9303x + 578.11$; $R^2 = 0.8175$

ALL: y = $0.0169x^2 - 5.6105x + 619.73$; $R^2 = 0.825$

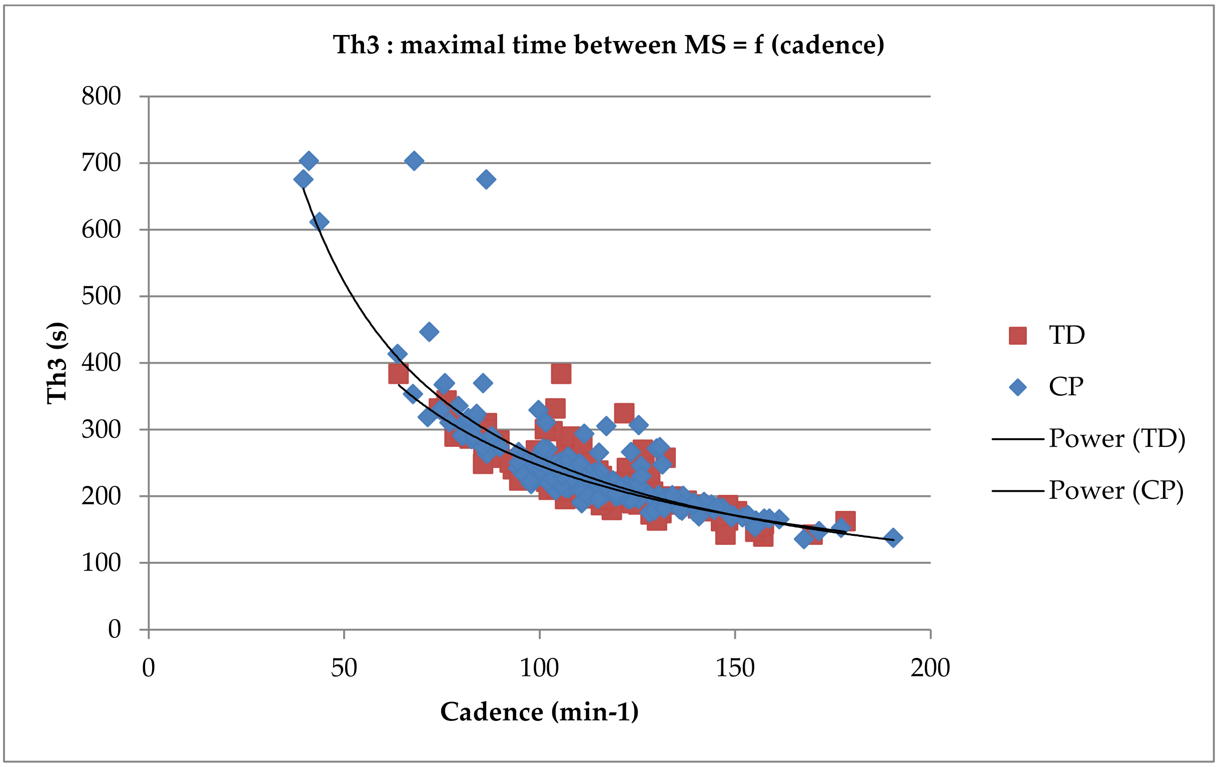

TD : y = $15116x^{-0.894}$; $R^2 = 0.7861$

CP : y = $27637x^{-1.015}$; R^2 = 0.6785

ALL : y = $23558x^{-0.984}$; R^2 = 0.7523

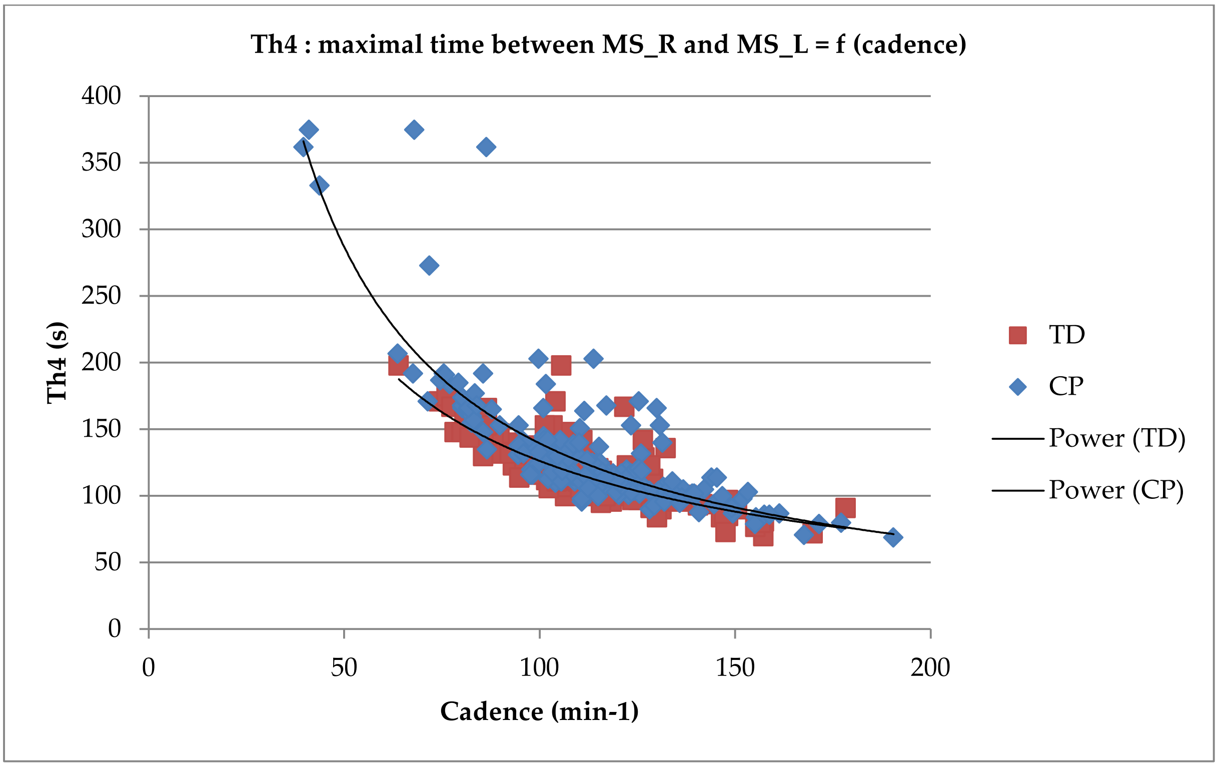

TD : y = $7442.2x^{-0.885}$; R^2 = 0.7379

CP : y = $16873x^{-1.042}$; R^2 = 0.6565

ALL : y = $14029x^{-1.011}$; R^2 = 0.7017
